# Influences of body protein and body fat on oestrus of yearling caribou

**DOI:** 10.1093/conphys/coag082

**Published:** 2026-10-15

**Authors:** Kristin Denryter

**Affiliations:** Foraging Ecology and Wildlife Nutritional Analysis Lab, Alaska Department of Fish and Game, 1801 S Margaret Dr, Ste 4, Palmer, AK 99645, USA

**Keywords:** Breeding, fecundity, fertility, ovulation, progestagens, sex hormone, steroid hormone

## Abstract

Age of primiparity is a critical demographic parameter that influences lifetime reproductive success and fitness. Caribou (*Rangifer tarandus*) exhibit variation in age of primiparity but generally are considered sexually immature (i.e. do not ovulate or achieve pregnancy) until they are ≥2.5 years old, unless nutrition is exceptional. Using captive, female, yearling caribou, I tested the exceptional nutrition hypothesis, focusing on ovulation because caribou were not permitted to breed. I quantified faecal progesterone concentrations throughout the breeding season and compared those to estimates of body mass, body fat and body protein of caribou. Body mass of yearling caribou ranged from 90.9 to 122.7 kg, body fat varied almost 5-fold (6.3–31.1%) and body protein ranged from 13.7 to 21.2 kg. Faecal progesterone ranged from 89 to 3334 ng/mg (673 ± 13; mean ± SE). Despite highly variable nutritional condition, all *n* = 11 yearlings exhibited oestrus. Mean date of first oestrus among yearling females was 22 October (±3 days) and caribou initiated oestrus 3.8 days earlier for every 1 kg increase in body protein. Yearling caribou experienced 3–9 (5.6 ± 0.7) oestrous cycles and for every 1% point increase in ingesta-free body fat, number of oestrous cycles between October and March increased by 0.3. Yearlings ovulated even in relatively marginal nutritional condition, indicating exceptional nutrition was not required for ovulation. Population assessments assuming yearling caribou are largely sexually immature may require additional scrutiny or contextualization, particularly if they are used to gauge nutritional status of populations to inform alternative conservation and management strategies.

## Introduction

When and how often organisms reproduce is an evolved trait and a key component of fitness. In iteroparous organisms, age of first reproduction (primiparity) is a critical demographic parameter that influences lifetime reproductive success and hence fitness (Cole, 1954; Stearns, 1992). Evolved age of primiparity should reflect selection to maximize lifetime reproductive success through an optimal allocation of limited resources among competing demands for growth, survival and reproduction (Cody, 1966; Demetrius, 1975; Garnier *et al.*, 2016). Although early breeding creates the potential for greater lifetime reproductive success and population productivity, breeding too young can be maladaptive (Descamps *et al.*, 2006). For example, individuals that reproduce prior to achieving asymptotic body size may forfeit additional somatic growth, which could be disadvantageous over time (Stamps *et al.*, 1998), and they or their offspring may face higher mortality (Ozoga and Verme, 1986; Blomquist, 2009). Delaying primiparity, however, may result in lost opportunities for reproduction and reductions in fitness, particularly if an individual dies before it reproduces (Blomquist, 2009). Thus, a younger age of primiparity should be selected for when individuals have an elevated risk of death prior to reproduction. In contrast, an older age of primiparity should be selected for when future reproductive success outweighs lost reproductive opportunities associated with delaying primiparity (Partridge and Harvey, 1988; Martin and Festa-Bianchet, 2012). Through effects on lifetime reproduction, age of primiparity can affect fitness and population rates of change (Swain *et al.*, 2007; Blomquist, 2009), making it an important parameter in monitoring population growth and recovery.

Although selective pressures result in an evolved or ‘fundamental’ age of primiparity—the youngest age at which an animal is capable of reproduction—animals may not always achieve their first reproduction at that age, resulting in a different ‘realized’ age of primiparity. Caribou (*Rangifer tarandus*) exemplify the difference between fundamental and realized age of primiparity because the age at which they reach sexual maturity varies (Dauphine, 1976; Thomas and Barry, 1990; Davis *et al.*, 1991; Ouellet *et al.*, 1997). Prevailing assumptions are that most female caribou give birth to their first calf at ≥3 years old (Whitten, 1991; Cameron and Ver Hoef, 1994; Adams and Dale, 1998; Boertje *et al.*, 2012), thus having reached sexual maturity at ~2.5 years old. Female yearling caribou, however, have been documented giving birth to their first calf at 2 years old (Thomas, 1982; Ouellet *et al.*, 1997; Adams *et al.*, 2019), which suggests a younger fundamental age of primiparity than often is observed. Differences between the fundamental and realized age of reproduction may be due to nutrition, as pregnancy in yearling caribou is thought to occur only when nutrition is exceptional (Reimers, 1983; Davis *et al.*, 1991; Cameron and Ver Hoef, 1994; Boertje *et al.*, 2012). The exceptional nutrition hypothesis has not been tested rigorously, but because age of primiparity often is used to index nutritional status of caribou populations (Wittmer *et al.*, 2005; Courtois *et al.*, 2007; Boertje *et al.*, 2012), it is important to ensure assumptions about relationships between nutrition and reproduction are accurate.

Nutrition influences virtually every aspect of reproduction, including age of primiparity, oestrus and ovulation, gestation and foetal growth, and timing of parturition (Reimers, 1983; Crête *et al.*, 1993; Chan-McLeod *et al.*, 1994; Valkenburg *et al.*, 2003; Post and Forchhammer, 2008; Proffitt *et al.*, 2021). Individuals in better nutritional condition typically have higher levels of progesterone (Folman *et al.*, 1973; Nasser *et al.*, 2011; Rivera *et al.*, 2011; Denicol *et al.*, 2012; Pursley and Martins, 2012), breed earlier and have higher pregnancy rates than animals in poorer condition (Cameron and Ver Hoef, 1994; Gerhart *et al.*, 1997a; Adams and Dale, 1998; Cook *et al.*, 2004). The influence of nutrition on reproductive processes, however, is more nuanced than often is appreciated (Denryter *et al.*, 2026a). For example, body fat and body mass do not directly regulate fertility or pregnancy; rather, their role in fertility may be related to an animal’s overall energy balance (Bronson and Manning, 1991; Gerhart *et al.*, 1997b). In yearling caribou, it also remains unclear whether nutrition constrains reproduction by inducing failures in ovulation, essentially eliminating reproductive potential, or whether failures occur at conception or gestation. Further, the potential role of body protein in reproduction is not as well understood, with the exception of maternal protein reserves supporting foetal growth (Barboza and Parker, 2006). Nonetheless, protein contributes to overall energy balance and hence could influence fertility. Protein may be especially important to reproductive processes in juveniles because they generally have smaller stores of body fat than adults, as they prioritize deposition of lean mass over fat (Adamczewski *et al.*, 1987; Gerhart *et al.*, 1996).

Caribou and reindeer are seasonal breeders. The breeding season occurs in autumn, with females experiencing oestrus ~3 weeks after shedding antler velvet (McEwan and Whitehead, 1972). Many hormones have key roles in reproduction, but the rapid rise in luteinizing hormone causes a follicle to rupture and release an ovum (ovulation), beginning the oestrous cycle. After ovulation, females experience a ~24-h period of sexual receptivity, and the ruptured follicle becomes the corpus luteum. The corpus luteum produces most of the progesterone required for establishing and maintaining pregnancy, and progesterone levels rise rapidly once production begins (Asher, 2011). If an ovum is not fertilized, the corpus luteum regresses ~15 days after ovulation, and progesterone production and levels drop rapidly. The subsequent nadir in progesterone levels occurs concurrently with a rise in luteinizing hormone levels, causing the next ovulation and oestrous cycle. Thus, for females that do not conceive, an oestrous cycle can be tracked by monitoring faecal progesterone levels and identifying consecutive nadirs (Yamauchi *et al.*, 1997). The predictable changes in progesterone, and the fact that it is shed in faeces, make it a useful, non-invasive tool for tracking oestrus in wildlife (Cook *et al.*, 2001b; Matsuura *et al.*, 2004; Schwarzenberger, 2007; Brown, 2018; Sontakke, 2018).

To evaluate whether nutrition constrains reproduction in female yearling caribou, I determined whether caribou experienced oestrus using faecal progesterone and compared characteristics of oestrus with body mass, body fat and body protein (as indicators of nutrition). Caribou were not permitted to breed, so I focused only on ovulation and oestrus as the potential nutritional bottleneck for reproduction in yearlings. Specifically, I aimed to test the hypothesis that onset, duration and number of oestrous cycles in a season were sensitive to nutrition. I predicted that yearling caribou in better nutritional condition would have higher concentrations of faecal progesterone, initiate oestrus earlier and have shorter and more oestrous cycles throughout the autumn–winter season than caribou in poorer condition. I predicted that body protein (as a reserve of both energy and protein) would have a stronger influence than body fat (a reserve of energy) on concentrations of faecal progesterone and initiation, duration and number of oestrous cycles among yearlings. Additionally, I aimed to test the exceptional nutrition hypothesis for sexual maturity (ovulation, oestrus) in yearlings. If exceptional nutrition was required for sexual maturity, I predicted that only animals in ‘good’ or ‘excellent’ [i.e. >7% body fat (Gunn and Nixon, 2008)] nutritional condition would ovulate and experience oestrus.

## Materials and methods

All study protocols were approved by the Institutional Animal Care and Use Committee (Protocols 0110-2022-02, 0118-2023-013, 0110-2024-09, 0110-2024-048). Caribou in this study were born in the wild, captured as neonates and hand-reared in captivity in 2022 and 2023. After weaning, caribou were housed in a 4-ha forested paddock where they were maintained on a pelleted ration (Reindeer D-Ration, Alaska Mill and Feed, Anchorage, AK) fed *ad libitum*, had access to pasture (*Bromus* sp., *Poa* sp., *Calamagrostis* sp.), tree cover and a lake. Caribou received freshly clipped browse (primarily *Salix* spp. and *Betula neoalaskana*) during the growing season and dried lichens (*Cetraria* spp., *Cladonia* spp., *Flavocetraria* spp., *Bryoria* spp., *Usnea* spp.) year-round. Browse and lichen species were chosen because they were identified as selected forages for caribou in previous work (Denryter *et al.*, 2017). During winter, animals were provided water in stock tanks with tank heaters and had access to snow as another source of water. Additional information on animal husbandry is provided elsewhere (Denryter and Beckmen, 2024; Denryter, 2026; Denryter *et al.*, 2026b).

As calves, caribou were trained to stand on a platform scale (S3 Weigh Scale Indicator, Tru-Test, Auckland, New Zealand; MP 800 load bars, Datamars Livestock, Temple, TX) for determination of body mass. Caribou were not trained for palpation of body condition scores, despite efforts (presumably because they are prey animals and touching near the back end is associated with actions of a predator). Thus, caribou had to be immobilized for manual palpation and ultrasonography along with routine hoof trimming, deworming, vaccination and removal of hard antlers (required for human handler and animal safety) in autumn (October). I immobilized caribou by hand-injecting 35–80 mg xylazine (Rompun 100 mg/ml, Dechra Veterinary Products, Overland Park, KS, USA) into a large muscle. I reversed xylazine with a minimum of 1 mg of atipamezole (Atipamezole Base in AQ vehicle, 25 mg/ml, Wedgewood Pharmacy, Swedesboro, NJ, USA) per 5 mg xylazine given intramuscularly, though sometimes an additional half dose was given intravenously if caribou did not reverse adequately within 5 min of the initial administration of atipamezole.

I collected body mass, body fat and body protein of caribou in early autumn, coinciding with the onset of the breeding season in October. To estimate body fat and body protein of yearling caribou in early October, I used techniques of Cook *et al.* (2021) developed and validated specifically for caribou in a whole-carcass homogenization study (Cook *et al.*, 2021). As with other body composition studies for wild ungulates (Stephenson *et al.*, 1998, 2020; Cook *et al.*, 2001a, 2010), the caribou study was validated for use across a wide range of nutritional condition and body sizes, regardless of sex, provided body fat is scaled for body mass, including for animals within the range of body size in this study. Prior to using these techniques, I received training from one of the co-authors of the whole-carcass homogenization study and I was the sole observer for collection of body fat and body protein data in this study. I used an ultrasound (Ibex-Pro, E.I. Medical Imaging, Loveland, CO) and a linear 5–8 mHz transducer, with a scanning depth of up to 12 cm, to measure the maximum thickness of rump fat (both years) and (in 2024 only) the loin muscle and fat over the loin muscle (if present) to the nearest 0.1 cm. Maximum thickness of rump fat was measured over the rump cranial to the tuber ischium. Loin muscle thickness was measured between the 12th and 13th ribs along the spine and any loin fat was measured directly over the loin muscle along the spine. I did not measure loin muscle thickness in 2023, but field studies with free-ranging caribou between 2023 and 2024 suggested it may be more important than previously thought (Denryter and Crouse, 2025a) and I began collecting it in 2024. In addition to collecting ultrasound measurements of fat and muscle, I also assigned a body condition score using manual palpation techniques (Cook *et al.*, 2021a). I estimated ingesta-free body fat (IFBF) using equations from Cook *et al*. (2021a) for scaled(maxfat + loinfat) and scaledLIVINDEXmaxfat + loinfat and estimated body protein from the loin muscle measurement, if available, otherwise I estimated body protein using the equation for body protein as a function of body mass. In accordance with validated equations for caribou (Cook *et al.*, 2021a), I scaled body fat, but not body protein, for body mass using mass collected within 3 days of ultrasonography for body fat.

In 2023 and 2024, research assistants and I collected faeces from yearling female caribou. In both years, female yearling caribou were physically separated from male caribou and never offered an opportunity to breed, though they were able to see and smell one another. After female caribou shed velvet from their antlers in mid- to late September, research assistants and I monitored them daily in October–November 2023 and September–March 2024–25 and, after observing defecation, collected at least five faecal pellets from each individual. Although not systematically, we recorded behavioural observations that were potentially indicative of oestrus, including dilation of the preorbital gland and raising of the tail (McEwan and Whitehead, 1972); pelvic tilts; being mounted by other females; rubbing on handlers, conspecifics or inanimate objects; showing greater than usual interest in a caribou bull; and sexual interest from the bull toward a specific female.

Faecal samples were stored in a −20°C freezer until they were freeze-dried in a lyophilizer (Harvest Right, LLC., Model HR9000-AL, UT, USA) for a minimum of 24 h; some large samples required additional freeze-drying time. After freeze-drying, faecal samples were homogenized to a fine powder using an electric coffee grinder that was cleaned with an air duster and wiped down with ethanol between samples. Freeze-dried, ground faecal samples were stored in airtight glass vials in a −20°C freezer until they were prepared for progesterone extraction.

### Progesterone extraction and assay techniques

Approximately 0.1 g of faecal powder was placed into a 2 ml cryovial to which 1.0 ml of 99.8% MeOH was added. Cryovials were agitated on a plate shaker (VWR Microplate Shaker 12620-926, Radnor, PA) at 900 Hz for 30 min and then transferred to a centrifuge for 20 min at 13 300 rpm (Heraeus Pico 17, ThermoScientific, Waltham, MA). The supernatant was decanted into a new cryovial, which was stored in a freezer until further analysis.

Prior to analysing caribou samples for progesterone concentrations, it was determined that a 1:144 dilution provided a binding percentage between 30 and 70%, as needed for the assay. After diluting samples with assay buffer, progesterone concentration was determined using a (species independent) DetectX progesterone ELISA enzyme immunoassay kit (K025-H5; Arbor Assays, Ann Arbor, MI), which was validated for accuracy and parallelism (Supplementary Information 1). For accuracy validation, a known amount of progesterone was added to a sample and then the observed and expected concentrations were compared. Parallelism validations allowed for evaluation of whether the kit detected progesterone in the sample proportionately to known standard concentrations (Supplementary Information 1). Validations were performed by creating a mixture of 10 random samples of 30 μl each in one test tube for a total volume of 300 μl. From this mixture, 20 μl of sample was pipetted into a test tube for accuracy testing (accuracy pool) and 40 μl into a test tube for parallelism testing (parallelism pool). Both test tubes evaporated passively overnight in a fume hood and the following day were reconstituted with 20 μl of 96% ethanol (Fisher Bioreagents, Fair Lawn, New Jersey, USA), vortexed for 20 s, and combined with 750 μl of assay buffer from the assay kit. For accuracy testing, 60 μl of the accuracy pool was mixed with 60 μl from each of seven standards from the assay kit for a total of eight test tubes (accuracy pool, pool + standard 1, …, pool + standard 7). For parallelism testing, the parallelism pool was serially diluted to create six additional dilutions of 1:2, 1:4, 1:8, 1:16, 1:32 and 1:64 (Ajó *et al.*, 2022). After determining progesterone levels using the assay kit, the accuracy validation was evaluated by calculating and graphing the expected versus observed values, with an acceptable slope being between 0.7 and 1.3 (Ajó *et al.*, 2022) (Fig. S1.1, Supplementary Information 1). For parallelism validation, the serially diluted samples were plotted with standards (Fig. S1.2, Supplementary Information 1). If slopes of serially diluted samples and standards were within 10% of one another, then parallelism validation was considered successful and indicated that progesterone was present in the samples (Grotjan and Keel, 1996). Assay plates were washed using an Agilent 50 TS Plate Washer (Agilent Technologies, Inc., Santa Clara, CA) and read for absorbance at 450 nm using a BioTek 800 TS Microplate Reader (BioTek Instruments, Inc., Winooski, VT). Following validation of faecal progesterone concentrations with this kit, samples were assayed and all standards, controls and samples were run in duplicate. The intra-assay coefficient of variation (CV) was calculated to assess the precision of each run (within a plate) using the CV from each sample (i.e. the sum of all sample CVs on a plate divided by the number of samples on the plate). Assays were repeated on samples with a CV ≥ 10 (Supplementary Information 1).

### Statistical analyses

I conducted all statistical analyses in STATA 14 (StataCorp, College Station, TX). Although there were repeated measures of progesterone on individual caribou, I did not have repeated measures of body mass, body fat or body protein throughout winter, and each individual only had one value for the response variables of mean faecal progesterone concentration, peak faecal progesterone concentration, date of first oestrus and number of oestrous cycles, so I used fixed-effects linear regression for those analyses. I regressed each response variable in separate models against body mass, body fat and body protein to evaluate whether there was an effect of nutrition on progesterone. To determine the number of days in an oestrous cycle, I calculated the difference between two consecutive nadirs in faecal progesterone concentrations for each individual (Cook *et al.*, 2001; Yamauchi *et al.*, 1997). I evaluated candidate models using Akaike’s Information Criterion corrected for small sample size (AICc) and determined the most parsimonious model as the one having the lowest AICc value (Anderson and Burnham, 2002; Burnham and Anderson, 2002). Because AICc is sensitive to *n*, only models using the same number of observations were compared with one another.

## Results

Body mass of yearling caribou ranged from 90.9–122.7 kg (105.7 ± 3.5 kg, mean ± SE). Body fat of yearling caribou (*n* = 11) ranged from 6.3–31.1% (15.6 ± 2.8%). Body protein of yearling caribou ranged from 13.7–21.2 kg (17.1 ± 0.8 kg). Body fat, body protein and body mass were strongly correlated with one another (Table 1, Supplemental Information 2).

**Table 1:** Relations between body fat, body mass and body protein of female yearling caribou in captivity in southcentral Alaska in autumn 2023–24

**Response variable**	**Predictor variable**	***F*(1, 9)**	***P***	***r*** ^**2**^	**Adj. *r*** ^**2**^
Body fat	Body protein	36.31	<0.001	0.801	0.779
Body fat	Body mass	62.12	<0.001	0.873	0.859
Body protein	Body mass	344.11	<0.001	0.975	0.972

Research assistants and I collected a total of 1585 faecal samples from yearling female caribou, including 154 faecal samples from *n* = 3 caribou in 2023 and 1431 faecal samples from *n* = 8 caribou in 2024–25. In 2023, we collected 48–53 (51 ± 2; mean ± SE) faecal samples from individual caribou, and in 2024–25 we collected 168–188 (178 ± 2). We missed collections on >1 but <3 consecutive days of faecal collections per individual caribou three times in 2023 and once in 2024–25.

Progesterone concentration in faecal samples from *n* = 11 female yearling caribou ranged from 89–3334 ng/mg (673 ± 13), varied among individuals (Fig. 1, Table 2), and indicated that all yearling females (*n* = 11) experienced at least 1 oestrous cycle (Fig. 2, Supplemental Information 2). The most parsimonious model explaining mean faecal progesterone concentration included body protein, though the model with body mass also was competitive (Table 3). In *n* = 11 female yearling caribou, faecal progesterone increased 51 ng/mg for every 1 kg increase in body protein (Fig. 3, Table 4, *F*(1, 9) = 5.97, *P* = 0.037, *r*^2^ = 0.399, Adj. *r*^2^ = 0.332). Peak concentration of faecal progesterone was not related to body mass, body fat or body protein of caribou (all *P* ≥ 0.294, Supplemental Information 2).

**Figure 1:**
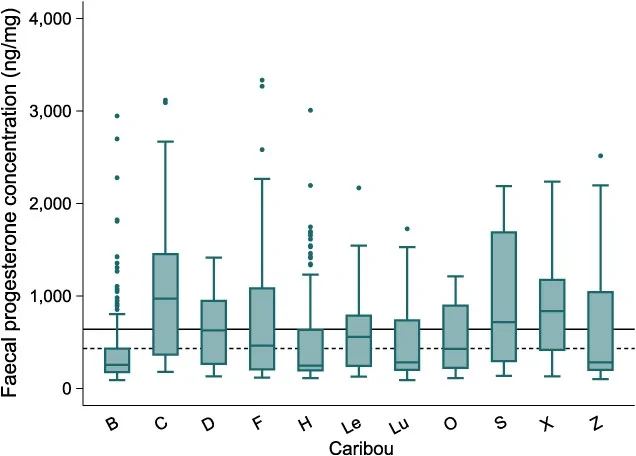
Progesterone concentrations in faecal samples from female yearling caribou in captivity in southcentral Alaska in autumn 2023 and autumn–winter 2024–25. F, O and S were yearlings in 2023 and all others were yearlings in 2024–25. Sample sizes by animal are provided in Table 2. The solid horizontal line shows the mean (639) of all faecal progesterone concentrations for all animals and the dashed horizontal line shows the median (433). The centre line of each boxplot represents the median of faecal progesterone, the bottom line of the box represents the first quartile of faecal progesterone and the top line of the box represents the third quartile of faecal progesterone. The bottom and top whiskers, respectively, are equal to the first quartile minus 1.5 times the interquartile range and the third quartile plus 1.5 times the interquartile range. Individual circles are outliers of faecal progesterone for each individual caribou.

**Table 2:** Progesterone concentrations in faecal samples from female yearling caribou in captivity in southcentral Alaska in autumn 2023–24 and winter 2025; animals F, O and S were yearlings in 2023 and all others were yearlings in 2024–25

**Animal**	**Mean**	**SE**	**Median**	**Min**	**Max**	***N***
B	410	31	254	90	2948	188
C	994	48	970	179	3117	183
D	640	27	628	131	1416	186
F	805	112	463	118	3334	53
H	488	35	247	111	3009	183
LE	558	26	559	128	2170	175
LU	467	27	281	89	1727	175
O	556	49	428	111	1212	53
S	981	101	718	135	2188	48
X	820	35	836	131	2238	173
Z	627	45	282	100	2517	168

**Figure 2:**
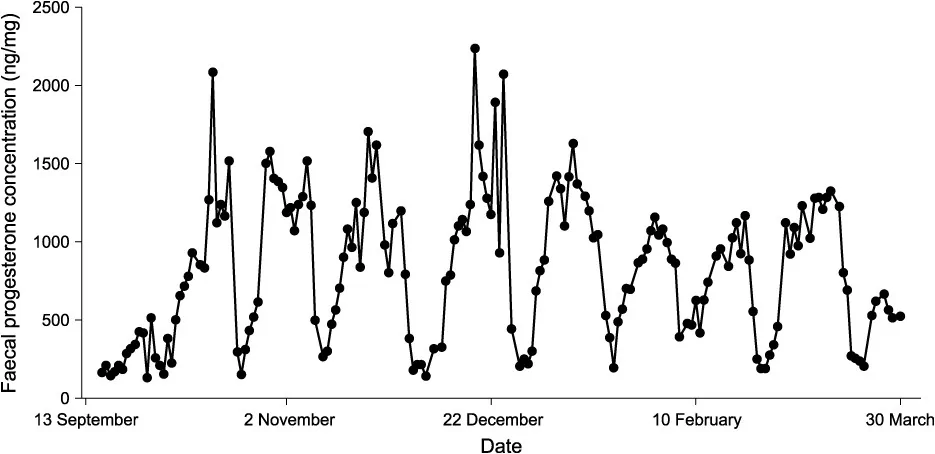
Progesterone concentrations in faecal samples from *n* = 1 female yearling caribou (X) in captivity in southcentral Alaska throughout autumn–winter 2024–25 showing 8 complete oestrous cycles and the initiation of a 9th oestrous cycle. Initiation of oestrus is exhibited by a nadir in progesterone levels and one oestrous cycle comprising the time between two consecutive nadirs. Oestrous cycles of other caribou are provided in Supplemental Information 2, available online.

**Table 3:** Ranks of candidate models for mean faecal progesterone concentration, date of first oestrus and number of oestrous cycles of female yearling caribou in captivity in southcentral Alaska in autumn 2023 (mean progesterone only) and autumn–winter 2024–25

**Model**	***K***	***n***	**LL**	**AICc**	**ΔAICc**	***w*** _***i***_	**Relative likelihood**
Mean progesterone ~ body protein	2	11	−70.736	146.972	0.000	0.610	1.000
Mean progesterone ~ body mass	2	11	−71.481	148.462	1.490	0.290	0.475
Mean progesterone ~ body fat	2	11	−72.543	150.585	3.613	0.100	0.164
							
First oestrus date ~ body protein	2	8	−20.662	47.725	0.000	0.865	1.000
First oestrus date ~ body mass	2	8	−22.550	51.499	3.775	0.131	0.151
First oestrus date ~ body fat	2	8	−25.976	58.351	10.627	0.004	0.005
							
Number of cycles ~ body fat	2	8	−11.939	30.279	0.000	0.608	1.000
Number of cycles ~ body protein	2	8	−12.838	32.076	1.797	0.247	0.407
Number of cycles ~ body mass	2	8	−13.372	33.145	2.866	0.145	0.239

**Figure 3:**
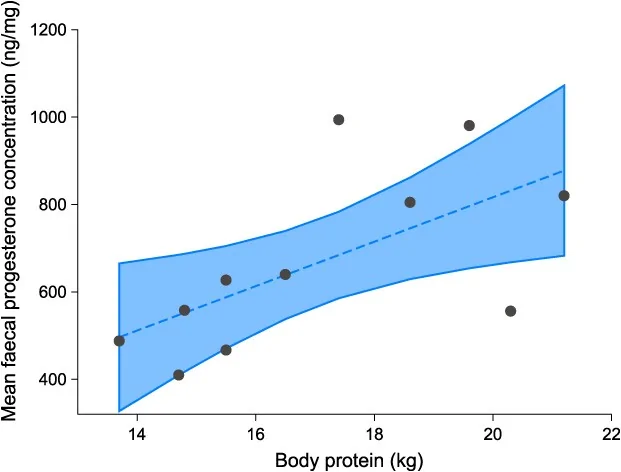
Mean faecal progesterone concentration as a function of body protein in *n* = 11 female yearling caribou in captivity in southcentral Alaska in autumn 2023 and autumn and winter 2024–25. The dashed line indicates the predicted value of faecal progesterone and the shaded areas represent 95% CIs from the most parsimonious regression model.

**Table 4:** Results from top models, as indicated by Akaike Information Criteria corrected for small sample size (Table 3), for mean faecal progesterone concentration, first oestrus date and number of oestrous cycles of female yearling caribou in captivity in southcentral Alaska in autumn 2023 (mean progesterone only) and autumn–winter 2024–25

**Model**	**Parameter**	**Coef.**	**SE**	***t***	***P***	**95% CI**	**85% CI**
Mean progesterone ~ body protein	Body protein	50.8	20.8	2.44	0.037	3.78–97.9	18.1–83.6
	Intercept	−200	359	−0.560	0.591	−1011–611	−764–364
							
First oestrus date ~ body protein	Body protein	−3.82	0.60	−6.36	0.001	−5.29–(−2.35)	−4.81–(−2.83)
	Intercept	358	9.79	36.6	<0.001	334–382	362–374
							
Number of cycles ~ body fat	Body fat	0.24	0.07	3.48	0.013	0.073–0.416	0.123–0.360
	Intercept	2.75	0.90	3.04	0.023	0.533–4.96	1.25–4.24

Abbreviation: CI, confidence interval.

I could not confirm the date of the beginning of the first or last oestrous cycle in 2023, because animals were sampled for only two months that year, but the earliest a yearling female came into oestrus in 2024 was 2 October, with a mean onset of 22 October (±3 days). Initiation of the latest last oestrous cycle in 2025 was 22 March, with a mean onset of the last oestrous cycle of 11 February (±13 days). The first (earliest) and last (latest) oestrous cycles occurred in the same female (Fig. 2). The most parsimonious model explaining date of first oestrus included body protein, though the model with body mass was competitive (Table 3). Yearling caribou initiated oestrus 3.8 days earlier for every 1 kg increase in body protein (Fig. 4, Table 4, *F*(1, 6) = 40.43, *P* < 0.001, *r*^2^ = 0.871, Adj. *r*^2^ = 0.849).

**Figure 4:**
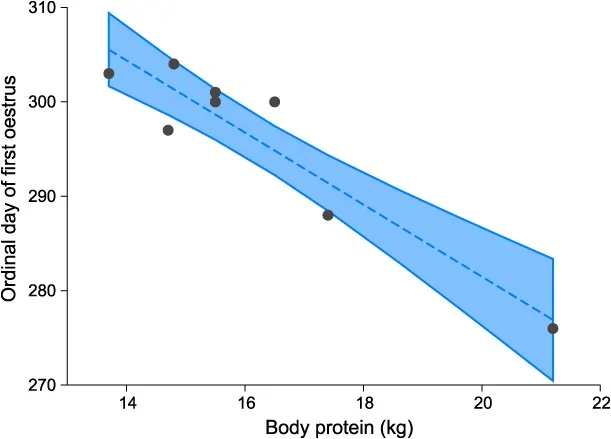
Date of initiation of first oestrus as a function of body protein in *n* = 8 female yearling caribou in captivity in southcentral Alaska in autumn 2024. Ordinal day 270 is 27 September and ordinal day 310 is 6 November. The dashed line indicates the predicted value of ordinal day of first oestrus and the shaded areas represent 95% CIs from the most parsimonious regression model.

The mean length of oestrous cycles in *n* = 8 yearling caribou was 22 ± 0.7 days, ranging in length from 11–33 days. Length of oestrous cycles was not related to body mass, body fat or body protein (all *P* ≥ 0.436, Supplemental Information 2). The mean number of oestrous cycles between September and March in yearling caribou was 5.6 ± 0.7 (*n* = 8) and ranged from 3–9 per individual. The most parsimonious model explaining number of oestrous cycles included body fat (Table 3). For every 1% point increase in IFBF, number of oestrous cycles between October and March increased by 0.3 (Fig. 5, Table 4, *F*(1, 6) = 16.13, *P* = 0.007, *r*^2^ = 0.729, Adj. *r*^2^ = 0.684).

**Figure 5:**
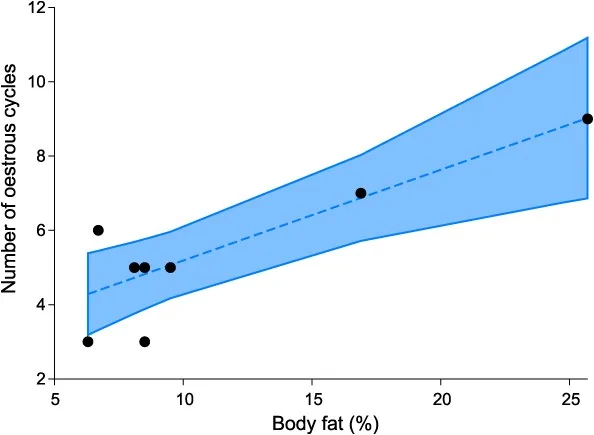
Number of oestrous cycles between October 2024 and March 2025 as a function of body fat in *n* = 8 female yearling caribou in captivity in southcentral Alaska in autumn 2024. The dashed line indicates the predicted number of oestrous cycles and the shaded areas represent 95% CIs from the most parsimonious regression model.

## Discussion

The age at which females of iteroparous species reach sexual maturity has important implications to fitness, population growth and conservation. The earlier a female can become pregnant, the greater her potential for lifetime reproduction and the sooner she can contribute to population growth, which is important for caribou populations that have declined throughout much of their range (Vors and Boyce, 2009). I tested the hypothesis that yearling caribou required exceptional nutrition to initiate oestrus—an indication of sexual maturity and a prerequisite for pregnancy. Across an almost 5-fold variation in body condition, all yearling females (*n* = 11) experienced at least 1 oestrous cycle, indicating they all possessed the potential to become pregnant as yearlings. Yearling caribou in better nutritional condition had higher faecal progesterone concentrations, initiated oestrus earlier and cycled more throughout autumn and winter than animals in poorer nutritional condition, supporting my hypothesis. In addition to changes in progesterone, research assistants and I observed (though not systematically) signs of oestrus in yearling caribou, with mounting by conspecific females, pelvic tilts, tail raises (with or without stimulation) and dilation of the preorbital gland on days females appeared to be in standing heat (oestrus). Additionally, caribou with 6.3 and 6.7% body fat—a level of nutrition considered ‘fair’ or ‘marginal’ (Cook *et al.*, 2004; Cook *et al.*, 2021; Gunn and Nixon, 2008) and below a suspected threshold for pregnancy (Crête and Huot, 1993)—experienced oestrus. I therefore reject the hypothesis that yearling caribou require exceptional nutrition to ovulate, but I could not determine whether there was a threshold of body fat (or body protein or body mass) needed for yearling caribou to ovulate.

Nutritional condition of yearlings influenced almost every aspect of oestrus, which may have been through effects on hormones including progesterone. Elevated progesterone levels were associated with increasing nutritional condition in caribou, consistent with findings for yaks (*Bos grunniens*) (Medhi *et al.*, 2018) and dairy cattle (*Bos taurus*) (Folman *et al.*, 1973). Progesterone production is energetically demanding and requires pregnenolone—a cholesterol metabolite also needed for synthesis of cortisol and other steroid hormones (Taraborrelli, 2015). When cholesterol is limited, progesterone synthesis may be diminished because production of cortisol may be prioritized, through a hypothesized phenomenon known as ‘pregnenolone steal’ (Taraborrelli, 2015). If pregnenolone steal was happening in caribou, it could explain the difference in progesterone levels relative to differences in nutritional condition. Greater levels of progesterone, owing to improved nutrition, may confer fitness benefits as they are associated with better quality oocytes (Pursley and Martins, 2012), higher rates of embryo survival (Kenny *et al.*, 2001) and greater reproductive success (Folman *et al.*, 1973). Likewise, lower progesterone levels during development of the corpus luteum negatively affect embryo quality (Nasser *et al.*, 2011; Rivera *et al.*, 2011) and probability of pregnancy (Shotton *et al.*, 1978; Denicol *et al.*, 2012).

I expected protein would be important to sexual maturity in yearling caribou because priority is given to deposition of lean mass rather than fat in juveniles (Adamczewski *et al.*, 1987; Gerhart *et al.*, 1996) and because caribou finance most foetal growth from maternal body protein (Barboza and Parker, 2008). Although this study did not provide a cause-and-effect relationship, there was an association between body protein and mean date of initiation of first oestrus (though body protein and body fat were strongly correlated, *r*^2^ = 0.801, so I caution against overinterpretation of the importance of body protein versus body fat in influencing oestrus). In domestic ruminants, loss of body protein can delay oestrous cyclicity, but the mechanism also may be related to body fat and energy balance (Robinson, 1996). Nonetheless, protein deficiencies may influence levels of reproductive hormones and hence ovarian function (Diskin and Kenny, 2016; Hayati *et al.*, 2021). Caribou in this study likely were not in a negative protein balance or protein deficiency but may still have been accreting body protein in autumn. If a threshold of body protein is needed to achieve sexual maturity and the hormone cascade to support oestrous cyclicity (Allrich, 1994), that may explain the negative relationship between body protein and onset of oestrus. Previous work posited that for animals having adequate energy for survival, protein may become a primary reproductive constraint (Barboza and Parker, 2008), but my findings suggest protein could be a reproductive constraint even when body fat is relatively low. Because this study focused on yearlings, which had not achieved asymptotic body size, it is possible that effects of protein were more pronounced than they would be in older females with more stable body protein that is preferentially conserved (Dauphine, 1971; Torbit *et al.*, 1985). Thus, body protein may be a better index of nutritional condition than body fat in young animals (Gerhart *et al.*, 1996). However, more work is needed to evaluate this hypothesis as relations between protein and fertility are complicated. For example, too much dietary protein can have detrimental effects on fertility (O’Callaghan and Boland, 1999), though these are unlikely to be an issue for caribou and other northern ungulates during the breeding season (Cook *et al.*, 2016; Denryter *et al.*, 2022).

Yearling caribou entering winter with more body fat experienced more oestrous cycles than those with less body fat. Cycling multiple times throughout winter is not abnormal for female ungulates that are not bred and has been reported in red deer (Jaczewski, 1989), moose (six cycles; Schwartz, 1992) and muskox (seven cycles; Rowell and Flood, 1988). In this study, females in better nutritional condition started cycling earlier and continued cycling for months after animals in poorer condition ceased cycling; however, this relationship was noisy for the thinnest females and there were few very fat animals for evaluating that relationship. The fattest females in this study were among the fattest reported in published literature, which is not surprising given the animals were maintained in captivity, provided a high-quality food *ad libitum* and likely had lower energy expenditures than free-ranging, migratory caribou. Nevertheless, despite *ad libitum* food, the caribou in this study had different appetites that resulted in an almost 5-fold difference in body fat. The thinnest caribou in this study were still approximately twice as fat as the thinnest caribou in the wild, which had <3% body fat (Cook *et al.*, 2021a; Denryter and Crouse, 2025b). The range of variation in body fat reported herein is not dissimilar from caribou populations in the wild (Cook *et al.*, 2021b; Denryter and Crouse, 2025a), except for the fattest animals herein (Supplementary Information 2). How body fat may have influenced number of oestrous cycles is not clear because a simple biological mechanism such as exhaustion of body fat reserves is inadequate by itself to explain the number of oestrous cycles. For example, the thinnest caribou gained 3.8 percentage points of body fat over winter and one of the intermediate animals gained 1.9 percentage points, but all others lost body fat over winter (Table S2.4, Supplementary Information 2). However, all females lost body protein over winter (range −3.3 to −0.2 kg), which could play a role in number of oestrous cycles through an influence on overall energy balance (I’Anson *et al.*, 1991). More work is needed to determine links between nutrition and number of oestrous cycles and these results should not be considered conclusive.

This study provided insights that inform our understanding of the reproductive potential of yearling caribou in general, but there are a few important caveats. First, I used an information-theoretic approach to evaluate models, which allows a set of competing candidate models to be ranked, but it does not necessarily provide a ‘right’ answer, just the most parsimonious answer among the options provided. My sample sizes were small, so it also is possible that future work may come to different conclusions. Second, all females were housed together, which may raise questions about pheromones influencing oestrus. Cycle-altering female pheromones have not been identified and there is scant evidence supporting hypotheses about female pheromones influencing ovulatory cycles, especially in non-rodent animals (Strassmann, 1999). One study in red deer suggested wild females may synchronize among each other when associated during rut (Iason and Guinness, 1985). If synchronization by pheromones was occurring owing to female association, it would not differ from conditions in the wild in highly gregarious ungulates that form large aggregations during the breeding season. Additionally, if females had been signalling each other through pheromones, I would have expected more synchrony in oestrous cycles than what occurred. I also did not allow caribou the opportunity to breed, so I cannot confirm whether ovulation necessarily correlates with pregnancy, though ovulation is a key limiting step in reproduction. Nonetheless, nutrition-related abortion is rare in cervids (Robinette and Gashwiler, 1950; Taber, 1953; Dauphine, 1976) and typically occurs under only the most extreme nutritional limitations (Thorne *et al.*, 1976; Cook *et al.*, 2002, 2013). Early embryonic mortality was suggested in caribou in one study (Russell *et al.*, 1998), but sample sizes (*n* = 3) were limited. Given the rarity of nutrition-related abortion in cervids and that caribou in this study were in adequate nutritional condition to carry a pregnancy to term, as compared to wild females (Denryter and Crouse, 2025a), there is no compelling evidence to indicate these caribou would not have achieved and maintained pregnancy had they been given the opportunity to breed. Also, the progesterone assay was species-independent, but validations were performed with caribou samples and caribou displayed oestrus behaviour at every nadir in progesterone, confirming the assay reflected changes in progesterone values of caribou oestrous cycles.

Although captive caribou in this study had *ad libitum* access to food, which differs from free-ranging caribou, appetite is entrained by photoperiod in northern cervids, which generally results in reductions in intake and growth overwinter in the wild and in captivity (Ryg and Jacobsen, 1982; Larsen *et al.*, 1985; Mesteig *et al.*, 2000; author, unpublished data). However, compensatory and state-dependent nutritional responses of caribou have been documented in captive and free-ranging caribou overwinter, which result in thinner animals gaining mass or fat over winter, whereas fatter caribou tend to lose fat over winter (Dale *et al.*, 2008; Couturier *et al.*, 2009; Cook *et al.*, 2021b; Denryter and Crouse, 2025a, 2025b). Caribou in this study also exhibited state-dependent changes in nutritional condition, despite being on an *ad libitum* diet (Table S2.4, Supplementary Information 2), suggesting strong physiological and environmental controls on appetite and fat dynamics persist in captivity. Thus, conditions in captivity may be more similar to conditions in the wild than one might expect.

For populations of caribou where age of primiparity or age-specific reproductive rates are used to index nutrition (Wittmer *et al.*, 2005; Courtois *et al.*, 2007; Boertje *et al.*, 2012), conservation and management success require understanding how nutrition and age-specific reproductive rates are linked. My findings suggest that the realized age of primiparity may be delayed when nutritional limitations are severe enough to delay ovulation. Whether depression of ovulation rates among yearlings is strong enough to meaningfully limit population growth will depend on the age structure of the population and requires understanding how age-specific reproductive and recruitment rates contribute to overall rates of population change. Increased rates of ovulation, however, do not necessarily equate to increases in recruitment, so future work that follows pregnant yearlings to determine recruitment rates will help fill this knowledge gap. Nonetheless, that 100% of yearling females in this study ovulated, including animals in marginal nutritional condition, suggests that nutrition need not be exceptional for ovulation and ovulation rates substantially <100% among yearling caribou may signal some level of nutritional limitation. High ovulation rates across a 5-fold difference in body fat suggest that reproductive potential may be greater in yearling caribou, even on a more marginal plane of nutrition, than previously thought. Thus, population assessments assuming yearling caribou are largely sexually immature may require additional scrutiny or contextualization, particularly if they are used to gauge nutritional status of populations to inform conservation and management strategies.

## Supplementary Material

Web_Material_coag082

## Data Availability

Data are available from the corresponding author upon reasonable request and execution of a data sharing agreement consistent with Alaska Statutes and Alaska Department of Fish and Game policy.

## References

[ref1] Adamczewski JZ, Gates CC, Hudson RJ (1987) Fat distribution and indices of carcass composition in Coats Island caribou (*Rangifer tarandus groenlandicus*). Can J Zool 65: 368–374. 10.1139/z87-057.

[ref2] Adams LG, Dale BW (1998) Reproductive performance of female Alaskan caribou. J Wildl Manage 62: 1184–1195. 10.2307/3801982.

[ref3] Adams LG, Farnell R, Oakley MP, Jung TS, Larocque LL, Lortie GM, Mclelland J, Reid ME, Roffler GH, Russell DE (2019) Evaluation of maternal penning to improve calf survival in the Chisana Caribou Herd. Wildl Monogr 204: 5–46. 10.1002/wmon.1044.

[ref4] Ajó AF, Hunt KE, Dillon D, Uhart M, Sironi M, Rowntree V, Buck CL (2022) Optimizing hormone extraction protocols for whale baleen: tackling questions of solvent: sample ratio and variation. Gen Comp Endocrinol 315: 113828. 10.1016/j.ygcen.2021.113828. 34058189

[ref5] Allrich RD (1994) Endocrine and neural control of estrus in dairy cows. J Dairy Sci 77: 2738–2744. 10.3168/jds.S0022-0302(94)77216-7. 7814742

[ref6] Anderson DR, Burnham KP (2002) Avoiding pitfalls when using information-theoretic methods. J Wildl Manage 66: 912–918. 10.2307/3803155.

[ref7] Asher GW (2011) Reproductive cycles of deer. Anim Reprod Sci 124: 170–175. 10.1016/j.anireprosci.2010.08.026. 20884138

[ref8] Barboza PS, Parker KL (2006) Body protein stores and isotopic indicators of N balance in female reindeer (*Rangifer tarandus*) during winter. Physiol Biochem Zool 79: 628–644. 10.1086/502811. 16691528

[ref9] Barboza PS, Parker KL (2008) Allocating protein to reproduction in arctic reindeer and caribou. Physiol Biochem Zool 81: 835–855. 10.1086/590414. 18702605

[ref10] Blomquist GE (2009) Trade-off between age of first reproduction and survival in a female primate. Biol Lett 5: 339–342. 10.1098/rsbl.2009.0009. PMC2679923 19324637

[ref11] Boertje RD, Gardner CL, Kellie KA, Taras BD (2012) Fortymile Caribou Herd: Increasing Numbers, Declining Nutrition, and Expanding Range. Alaska Department of Fish and Game, Division of Wildlife Conservation, Juneau, AK, USA.

[ref12] Bronson FH, Manning JM (1991) The energetic regulation of ovulation: a realistic role for body fat. Biol Reprod 44: 945–950. 10.1095/biolreprod44.6.945. 1873394

[ref13] Brown JL (2018) Comparative ovarian function and reproductive monitoring of endangered mammals. Theriogenology 109: 2–13. 10.1016/j.theriogenology.2017.12.004. 29249329

[ref14] Burnham KP, Anderson DR (2002) Model Selection and Multi-Model Inference: A Practical Information Theoretic Approach, Ed 2nd. Springer, New York

[ref15] Cameron RD, Ver Hoef JM (1994) Predicting parturition rate of caribou from autumn body mass. J Wildl Manage 58: 674–679. 10.2307/3809681.

[ref16] Chan-McLeod ACA, White RG, Holleman DF (1994) Effects of protein and energy-intake, body condition, and season on nutrient partitioning and milk production in caribou and reindeer. Can J Zool 72: 938–947. 10.1139/z94-127.

[ref17] Cody ML (1966) A general theory of clutch size. Evolution (N Y) 20: 174–184. 10.1111/j.1558-5646.1966.tb03353.x 28563630

[ref18] Cole LC (1954) The population consequences of life history phenomena. Q Rev Biol 29: 103–137. 10.1086/400074. 13177850

[ref19] Cook JG, Cook RC, Davis RW, Irwin LL (2016) Nutritional ecology of elk during summer and autumn in the Pacific Northwest. Wildl Monogr 195: 1–81. 10.1002/wmon.1020.

[ref20] Cook JG, Johnson BK, Cook RC, Riggs RA, Bryant LD, Irwin LL (2004) Effects of summer-autumn nutrition and parturition date on reproduction and survival of elk. Wildl Monogr 155: 1–61. 10.2193/0084-0173(2004)155[1:EOSNAP]2.0.CO;2.

[ref21] Cook JG, Kelly AP, Cook RC, Culling B, Culling D, Mclaren A, Larter NC, Watters M (2021b) Seasonal patterns in nutritional condition of caribou (*Rangifer tarandus*) in the southern Northwest Territories and northeastern British Columbia, Canada. Can J Zool 99: 845–858. 10.1139/cjz-2021-0057.

[ref22] Cook RC, Cook JG, Garrott RA, Irwin LL, Monfort SL (2002) Effects of diet and body condition on fecal progestagen excretion in elk. J Wildl Dis 38: 558–565. 10.7589/0090-3558-38.3.558. 12238373

[ref23] Cook RC, Cook JG, Murray DL, Zager P, Johnson BK, Gratson MW (2001a) Development of predictive models of nutritional condition for Rocky Mountain elk. J Wildl Manage 65: 973–987. 10.2307/3803046.

[ref24] Cook RC, Cook JG, Stephenson TR, Myers WL, McCorquodale SM, Vales DJ, Irwin LL, Hall PB, Spencer RD, Murphie SL et al. (2010) Revisions of rump fat and body scoring indices for deer, elk, and moose. J Wildl Manage 74: 880–896. 10.2193/2009-031.

[ref25] Cook RC, Cook JG, Vales DJ, Johnson BK, McCorquodale SM, Shipley LA, Riggs RA, Irwin LL, Murphie SL, Schoenecker KA et al. (2013) Regional and seasonal patterns of nutritional condition and reproduction in elk. Wildl Monogr 184: 1–45. 10.1002/wmon.1008.

[ref26] Cook RC, Crouse JA, Cook JG, Stephenson TR (2021a) Evaluating indices of nutritional condition for caribou (*Rangifer tarandus*): which are the most valuable and why? Can J Zool 99: 596–613. 10.1139/cjz-2020-0149.

[ref27] Cook RC, Murray DL, Cook JG, Zager P, Monfort SL (2001b) Nutritional influences on breeding dynamics in elk. Can J Zool 79: 845–853. 10.1139/z01-050.

[ref28] Courtois R, Ouellet J-P, Breton L, Gingras A, Dussault C (2007) Effects of forest disturbance on density, space use, and mortality of woodland caribou. Ecoscience 14: 491–498. 10.2980/1195-6860(2007)14[491:EOFDOD]2.0.CO;2.

[ref29] Couturier S, Côté SD, Huot J, Otto RD (2009) Body-condition dynamics in a northern ungulate gaining fat in winter. Can J Zool 87: 367–378. 10.1139/Z09-020.

[ref30] Crête M, Huot J (1993) Regulation of a large herd of migratory caribou: summer nutrition affects calf growth and body reserves of dams. Can J Zool 71: 2291–2296. 10.1139/z93-321.

[ref31] Crête M, Huot J, Nault R, Patenaude R (1993) Reproduction, growth and body composition of Riviere George caribou in captivity. Arctic 46: 189–196. 10.14430/arctic1343.

[ref32] Dale BW, Adams LG, Collins WB, Joly K, Valkenburg P, Tobey R (2008) Stochastic and compensatory effects limit persistence of variation in body mass of young caribou. J Mammal 89: 1130–1135. 10.1644/07-MAMM-A-137.1.

[ref33] Dauphine TC (1971) Physical variables as an index to condition in barren-ground caribou. Trans Northeast Sect Wildl Soc 28: 91–108.

[ref34] Dauphine TC (1976) Biology of the Kaminuriak population of barren-ground caribou Part 4. Can Wildl Serv Rep Ser 38: 1–71.

[ref35] Davis JL, Adams LG, Valkenburg P, Reed DJ (1991) Relationships between body weight, early puberty, and reproductive histories in central Alaskan caribou. In E Butler, SP Mahoney, eds, Proceedings of the 4th North American Caribou Workshop, St. Johns, NL, Canada, pp 115–142.

[ref36] Demetrius L (1975) Reproductive strategies and natural selection. Am Nat 109: 243–249. 10.1086/282994.

[ref37] Denicol AC, Lopes J, Mendonça LGD, Rivera FA, Guagnini F, Perez RV, Lima JR, Bruno RGS, Santos JEP, Chebel RC (2012) Low progesterone concentration during the development of the first follicular wave reduces pregnancy per insemination of lactating dairy cows. J Dairy Sci 95: 1794–1806. 10.3168/jds.2011-4650. 22459828

[ref38] Denryter K (2026) Heat stress constrains milk intake and growth potential of hand-reared caribou calves. J Therm Biol 139: 104486. 10.1016/j.jtherbio.2026.104486. 42143889

[ref39] Denryter K, Beckmen KB (2024) Suspected case of persistent thiamin deficiency in a hand-reared caribou calf. Zoo Biol 43: 580–584. 10.1002/zoo.21864. PMC11624623 39321035

[ref40] Denryter K, Cook RC, Cook JG, Parker KL (2017) Straight from the caribou’s (*Rangifer tarandus*) mouth: detailed observations of tame caribou reveal new insights into summer–autumn diets. Can J Zool 95: 81–94. 10.1139/cjz-2016-0114.

[ref41] Denryter K, Cook RC, Cook JG, Parker KL (2022) Animal-defined resources reveal nutritional inadequacies for woodland caribou during summer in northeastern British Columbia. J Wildl Manage 86: e22161.

[ref42] Denryter K, Crouse JA (2025a) Overwinter gains in body fat challenge assumptions about winter nutrition in northern ungulates. Ecosphere 16: e70507. 10.1002/ecs2.70507.

[ref43] Denryter K, Crouse JA (2025b) *Summary of Capture Efforts for Nutrition and Disease Studies of the Mulchatna Caribou Herd Capture Dates: February 18–26*, 2025. Alaska Department of Fish and Game, Juneau, AK, USA.

[ref44] Denryter K, Parker KL, White RG (2026a) Are nutritional limitations in northern ungulates paradoxical? Rangifer 46: 1–30. 10.7557/2.46.1.8081.

[ref45] Denryter K, Pryor J, Puype A (2026b) Physiological indicators of caribou calf health and compensatory responses to physiological perturbations. J Mammal 107: 714–725. 10.1093/jmammal/gyag051. PMC13416188 42529107

[ref46] Descamps S, Boutin S, Berteaux D, Gaillard J-M (2006) Best squirrels trade a long life for an early reproduction. Proc R Soc B Biol Sci 273: 2369–2374. 10.1098/rspb.2006.3588. PMC1636082 16928640

[ref47] Diskin MG, Kenny DA (2016) Managing the reproductive performance of beef cows. Theriogenology 86: 379–387. 10.1016/j.theriogenology.2016.04.052. 27180327

[ref49] Folman Y, Rosenberg M, Herz Z, Davidson M (1973) The relationship between plasma progesterone concentration and conception in post-partum dairy cows maintained on two levels of nutrition. J Reprod Fertil 34: 267–278. 10.1530/jrf.0.0340267. 4795377

[ref50] Garnier A, Gaillard J-M, Gauthier D, Besnard AA (2016) What shapes fitness costs of reproduction in long-lived iteroparous species? A case study on the Alpine ibex. Ecol Soc Am 97: 205–214. 10.1890/15-0014.1. 27008789

[ref51] Gerhart KL, Russell DE, Van De Wetering D, White RG, Cameron RD (1997a) Pregnancy of adult caribou (*Rangifer tarandus*): evidence for lactational infertility. J Zool 242: 17–30. 10.1111/j.1469-7998.1997.tb02926.x.

[ref52] Gerhart KL, White RG, Cameron RD, Russell DE (1996) Estimating fat content of caribou from body condition scores. J Wildl Manage 60: 713–718. 10.2307/3802369.

[ref53] Gerhart KL, White RG, Cameron RD, Russell DE, Van De Wetering D (1997b) Pregnancy rate as an indicator of nutritional status in *Rangifer*: implications of lactational infertility. Rangifer 17: 21–24. 10.7557/2.17.1.381.

[ref54] Grotjan HE, Keel BA (1996) Data interpretation and quality control. In EP Diamandis, TK Christopoulos, eds, Immunoassay. Academic Press, New York, pp. 51–93.

[ref55] Gunn A, Nixon W (2008) Rangifer Health & Body Condition Monitoring Manual. Circum Arctic Rangifer Monitoring and Assessment (CARMA) Network, Vancouver, BC, Canada.

[ref56] Hayati RN, Panjono, Irawan A (2021) Estrous signs and progesterone profile of Ongole Grade cows synchronized at different ages fed different level of dietary crude protein. Trop Anim Sci J 44: 16–23. 10.5398/tasj.2021.44.1.16.

[ref57] I’Anson H, Foster DL, Foxcroft GR, Booth PJ (1991) Nutrition and reproduction. In SR Milligan, ed, Oxford Reviews of Reproductive Biology 13. Oxford University Press, Oxford. 1845336

[ref58] Iason GR, Guinness FE (1985) Synchrony of oestrus and conception in red deer (*Cervus elaphus* L.). Anim Behav 33: 1169–1174. 10.1016/S0003-3472(85)80177-9.

[ref59] Jaczewski Z (1989) Reproduction in the red deer female and the effect of oestrogens on the antler cycle and behaviour. Acta Physiol Pol 40: 85–95. 2603745

[ref61] Kenny DA, Boland MP, Diskin MG, Sreenan JM (2001) Effect of pasture crude protein and fermentable energy supplementation on blood metabolite and progesterone concentrations and on embryo survival in heifers. Anim Sci 73: 501–511. 10.1017/S1357729800058471.

[ref62] Larsen TS, Nilsson NO, Blix AS (1985) Seasonal changes in lipogenesis and lipolysis in isolated adipocytes from Svalbard and Norwegian reindeer. Acta Physiol Scand Scand 123: 97–104. 10.1111/j.1748-1716.1985.tb07566.x. 3969836

[ref63] Martin JGA, Festa-Bianchet M (2012) Determinants and consequences of age of primiparity in bighorn ewes. Oikos 121: 752–760. 10.1111/j.1600-0706.2011.19962.x.

[ref64] Matsuura Y, Sasamoto Y, Sato K, Takahashi Y, Suzuki M, Ohtaishi N (2004) Monitoring ovarian cycle and conception by fecal progesterone analysis in sika deer. Ecol Res 19: 397–404. 10.1111/j.1440-1703.2004.00650.x.

[ref65] McEwan EH, Whitehead PE (1972) Reproduction in female reindeer and caribou. Can J Zool 50: 43–46. 10.1139/z72-008. 5058558

[ref66] Medhi D, Santra A, Paul V, Deb SM (2018) Effect of plane of nutrition on blood biochemical parameters and attainment of sexual maturity in growing yaks. Indian J Anim Sci 88: 470–473. 10.56093/ijans.v88i4.78870.

[ref67] Mesteig K, Tyler NJC, Blix AS (2000) Seasonal changes in heart rate and food intake in reindeer (*Rangifer tarandus tarandus*). Acta Physiol Scand 170: 145–151. 10.1046/j.1365-201x.2000.00767.x. 11114952

[ref69] Nasser LF, Sá Filho MF, Reis EL, Rezende CR, Mapletoft RJ, Bó GA, Baruselli PS (2011) Exogenous progesterone enhances ova and embryo quality following superstimulation of the first follicular wave in Nelore (*Bos indicus*) donors. Theriogenology 76: 320–327. 10.1016/j.theriogenology.2011.02.009. 21496903

[ref70] O’Callaghan D, Boland MP (1999) Nutritional effects on ovulation, embryo development and the establishment of pregnancy in ruminants. Anim Sci 68: 299–314. 10.1017/S1357729800050311.

[ref71] Ouellet J-P, Heard DC, Boutin S, Mulders R (1997) A comparison of caribou on two predator-free arctic islands. Can J Zool 17: 11–17.

[ref72] Ozoga JJ, Verme LJ (1986) Relation of maternal age to fawn-rearing success in white-tailed deer. J Wildl Manage 50: 480–486. 10.2307/3801110.

[ref73] Partridge L, Harvey PH (1988) The ecological context of life history evolution. Science. 241: 1449–1455. 10.1126/science.241.4872.1449. 17790040

[ref74] Post E, Forchhammer MC (2008) Climate change reduces reproductive success of an Arctic herbivore through trophic mismatch. Philos Trans R Soc Lond B Biol Sci 363: 2369–2375. 10.1098/rstb.2007.2207. PMC2606787 18006410

[ref75] Proffitt KM, Courtemanch AB, Dewey SR, Lowrey B, McWhirter DE, Monteith KL, Paterson JT, Rotella J, White PJ, Garrott RA (2021) Regional variability in pregnancy and survival rates of Rocky Mountain bighorn sheep. Ecosphere 12: e03410. 10.1002/ecs2.3410.

[ref76] Pursley JR, Martins JPN (2012) Impact of circulating concentrations of progesterone and antral age of the ovulatory follicle on fertility of high-producing lactating dairy cows. Reprod Fertil Dev 24: 267–271. 10.1071/RD11917 22394968

[ref77] Reimers E (1983) Reproduction in wild reindeer in Norway (*Rangifer tarandus*). Can J Zool 61: 211–217. 10.1139/z83-026.

[ref78] Rivera FA, Mendonça LGD, Lopes G, Santos JEP, Perez RV, Amstalden M, Correa-Calderón A, Chebel RC (2011) Reduced progesterone concentration during growth of the first follicular wave affects embryo quality but has no effect on embryo survival post transfer in lactating dairy cows. Reproduction 141: 333–342. 10.1530/REP-10-0375. 21177956

[ref79] Robinette WL, Gashwiler JS (1950) Breeding season, productivity, and fawning period of the mule deer in Utah. J Wildl Manage 14: 457–469. 10.2307/3797276.

[ref80] Robinson JJ (1996) Nutrition and reproduction. Anim Reprod Sci 42: 25–34. 10.1016/0378-4320(96)01526-6.

[ref81] Rowell JE, Flood PF (1988) Progesterone, oestradiol-17β and LH during the oestrus cycle of muskoxen (*Ovibos moschatus*). J Reprod Fertil 84: 117–122. 10.1530/jrf.0.0840117. 3184036

[ref82] Russell DE, Gerhart KL, White RG, Van De WD (1998) Detection of early pregnancy in caribou: evidence for embryonic mortality. J Wildl Manage 62: 1066–1075. 10.2307/3802559.

[ref83] Ryg M, Jacobsen E (1982) Seasonal changes in growth rate, feed intake, growth hormone, and thyroid hormones in young male reindeer (*Rangifer tarandus tarandus*). Can J Zool 60: 15–23. 10.1139/z82-002.

[ref84] Schwartz C (1992) Reproductive biology North American moose. Alces 28: 165–173.

[ref86] Schwarzenberger F (2007) The many uses of non-invasive faecal steroid monitoring in zoo and wildlife species. Int Zoo Yearb 41: 52–74. 10.1111/j.1748-1090.2007.00017.x.

[ref87] Shotton SM, Roy JHB, Pope GS (1978) Plasma progesterone concentrations from before puberty to after parturition in British Friesian heifers reared on high planes of nutrition and inseminated at their first oestrus. Anim Sci 27: 89–98. 10.1017/S0003356100035856.

[ref88] Sontakke SD (2018) Monitoring and controlling ovarian activities in wild ungulates. Theriogenology 109: 31–41. 10.1016/j.theriogenology.2017.12.008. 29254686

[ref89] Stamps JA, Mangel M, Phillips JA (1998) A new look at relationships between size at maturity and asymptotic size. Am Nat 152: 470–479. 10.1086/286183. 18811453

[ref90] Stearns SC (1992) The Evolution of Life Histories. Oxford University Press, Oxford.

[ref91] Stephenson TR, German DW, Cassirer EF, Walsh DP, Blum ME, Cox M, Stewart KM, Monteith KL (2020) Linking population performance to nutritional condition in an alpine ungulate. J Mammal 101: 1244–1256. 10.1093/jmammal/gyaa091. PMC7733374 33335453

[ref92] Stephenson TR, Hundertmark KJ, Schwartz CC, Van Ballenberghe V (1998) Predicting body fat and body mass in moose with ultrasonography. Can J Zool 76: 717–722. 10.1139/z97-248.

[ref93] Strassmann BI (1999) Menstrual synchrony pheromones: cause for doubt. Hum Reprod 14: 579–580. 10.1093/humrep/14.3.579. 10221677

[ref94] Swain DP, Sinclair AF, Hanson JM (2007) Evolutionary response to size-selective mortality in an exploited fish population. Proc R Soc B Biol Sci 274: 1015–1022. 10.1098/rspb.2006.0275. PMC2124474 17264058

[ref95] Taber RD (1953) Studies of black-tailed deer reproduction on three chaparral cover types. Calif Fish Game 39: 177–186.

[ref96] Taraborrelli S (2015) Physiology, production and action of progesterone. Acta Obstet Gynecol Scand 94: 8–16. 10.1111/aogs.12771. 26358238

[ref97] Thomas DC (1982) The relationship between fertility and fat reserves of Peary caribou. Can J Zool 60: 597–602. 10.1139/z82-089.

[ref98] Thomas DC, Barry SJ (1990) Age-specific fecundity of the Beverly herd of barren-ground caribou. Rangifer 10: 257–263.

[ref99] Thorne ET, Dean RE, Hepworth WG (1976) Nutrition during gestation in relation to successful reproduction in elk. J Wildl Manage 40: 330–335. 10.2307/3800433.

[ref100] Torbit SC, Carpenter LH, Swift DM, Alldredge AW (1985) Differential loss of fat and protein by mule deer during winter. J Wildl Manage 49: 80–85. 10.2307/3801849.

[ref101] Valkenburg P, Pitcher KW, Reed DJ, Becker EF, Dau JR, Larsen DN, Davis JL (1991) Density dependent responses in mandible length, calving date, and recruitment in three Alaskan caribou herds. Proceedings of the 4th North American Caribou Workshop, St John’s, NL, Canada, pp 288–298.

[ref102] Valkenburg P, Tobey RW, Dale BW, Scotton BD, Ver Hoef JM (2003) Body size of female calves and natality rates of known-aged females in two adjacent Alaskan caribou herds, and implications for management. Rangifer 23: 203–209. 10.7557/2.23.5.1702.

[ref104] Vors LS, Boyce MS (2009) Global declines of caribou and reindeer. Glob Chang Biol 15: 2626–2633. 10.1111/j.1365-2486.2009.01974.x.

[ref105] Whitten KR (1991) Reproductive rates of radio-collared females in the Porcupine Caribou Herd. Proceedings of the 4th North American Caribou Workshop, St John’s, Newfoundland, pp 174–178.

[ref106] Wittmer HU, Sinclair ARE, McLellan BN (2005) The role of predation in the decline and extirpation of woodland caribou. Oecologia 144: 257–267. 10.1007/s00442-005-0055-y. 15891849

[ref107] Yamauchi K, Hamasaki S, Takeuchi Y, Mori Y (1997) Assessment of reproductive status of sika deer by fecal steroid analysis. J Reprod Dev 43: 221–226. 10.1262/jrd.43.221.

