# Supplementary material for: Influences of body protein and body fat on oestrus of yearling caribou
Source: Conserv Physiol. 2026 Oct 15;14(1):coag082. doi: 10.1093/conphys/coag082 (PMC13674547; doi:10.1093/conphys/coag082)
Supplement: Supplementary file 1 [file web_material_coag082.zip › Supplementary Information 1-3.docx]

Denryter, K. 2026. Influences of body fat and body protein in oestrus and reproductive potential of yearling caribou. Conservation Physiology.

**Supplementary Information 1.** Detailed methods for progesterone assays and validation results.

Protocol for ELISA validations:

Table S1.1. Quantities of solutions and samples needed for progesterone assays in µL.

| Sample | Hormone | Extract | Assay buffer | Accuracy pool extract | Accuracy pool assay buffer | Parallelism pool extract | Parallelism pool assay buffer |
| --- | --- | --- | --- | --- | --- | --- | --- |
| Caribou faecal | progesterone | 20 | 1500 | 20 | 750 | 40 | 750 |

Start-up

1) Make a mixture of 10 random samples of 30 µL each
2) Pipet 20 µL into a test tube labeled “accuracy pool” and 40 µL into a test tube labeled “parallelism pool”
3) Let test tubes air dry in the fume hood overnight
4) The next day, reconstitute each tube with 20 µL ethanol and vortex them for at least 20 seconds
5) Immediately add 750 µL assay buffer to each tube

Accuracy

1) Label seven additional test tubes as “S1 + pool….S7 + pool”
2) Following assay kit instructions, create the seven standards
3) Combine 60 µL of each standard and 60 µL of the accuracy pool to the corresponding test tubes from Accuracy Step 1
4) Follow the assay kit instructions for preparing and analyzing the plate
5) Calculated expected vs observed
   a. Expected value of “S1 + pool” = standard 1 divided by 2 + accuracy pool value divided by 2
6) Plot expected vs observed and the slope of the line should be between 0.7 and 1.3 (Ajó et al., 2022)

Parallelism

1) Use “parallelism pool” test tube from Start-up Steps 1-5
2) Make tubes labeled 1:2, 1:4, 1:8, 1:16, 1:32, and 1:64
3) Fill each tube with 120 µL assay buffer
4) Add 120 µL from the parallelism pool tube to the 1:2 tube, pipette tip several times to mix, then pull up 120 µL and mix into the 1:4 tube and continue the process until the 1:64 tube is mixed
5) Follow the assay kit instructions for preparing and analyzing the plate
6) Plot the serially diluted pool concentrations with the standards and the slopes of the two lines should be within 10% of one another (Grotjan & Keel, 1996) as displayed in Fig. S1.2.

| Observed progesterone concentration (ng/mg) | Expected progesterone concentration (ng/mg) |
| --- | --- |
| 1889 | 1600 |
| 939.3 | 800 |
| 574.5 | 400 |
| 397.8 | 200 |
| 253 | 100 |
| 258.9 | 50 |
| 234.5 | 25 |

**Figure S1.1.** Example accuracy validation for assays of progesterone in faecal samples from captive, female, yearling caribou in southcentral Alaska. The slope of 0.951 is within the acceptable range of 0.7–1.3 for accuracy validation (Ajó et al., 2022).

%B/B0

| pg/mL | 4PL |
| --- | --- |
| 50 | 85.65 |
| 54.43 | 84.95 |
| 59.25 | 84.18 |
| 64.5 | 83.34 |
| 70.21 | 82.42 |
| 76.43 | 81.42 |
| 83.2 | 80.33 |
| 90.57 | 79.15 |
| 98.6 | 77.88 |
| 107.3 | 76.5 |
| 116.8 | 75.03 |
| 127.2 | 73.45 |
| 138.5 | 71.77 |
| 150.7 | 69.98 |
| 164.1 | 68.09 |
| 178.6 | 66.1 |
| 194.4 | 64.02 |
| 211.6 | 61.84 |
| 230.4 | 59.59 |
| 250.8 | 57.26 |
| 273 | 54.87 |
| 297.2 | 52.44 |
| 323.5 | 49.97 |
| 352.2 | 47.48 |
| 383.4 | 44.98 |
| 417.3 | 42.49 |
| 454.3 | 40.03 |
| 494.6 | 37.6 |
| 538.4 | 35.22 |
| 586 | 32.91 |
| 638 | 30.67 |
| 694.5 | 28.51 |
| 756 | 26.44 |
| 823 | 24.47 |
| 895.9 | 22.6 |
| 975.2 | 20.83 |
| 1062 | 19.16 |
| 1156 | 17.6 |
| 1258 | 16.15 |
| 1369 | 14.79 |
| 1491 | 13.54 |
| 1623 | 12.37 |
| 1767 | 11.3 |
| 1923 | 10.32 |
| 2093 | 9.41 |
| 2279 | 8.582 |
| 2481 | 7.824 |
| 2700 | 7.134 |
| 2940 | 6.504 |
| 3200 | 5.932 |

| pg/mL | Standard |
| --- | --- |
| 3200 | 7.854 |
| 3200 | 5.189 |
| 1600 | 11.08 |
| 1600 | 12.76 |
| 800 | 24.68 |
| 800 | 24.4 |
| 400 | 45.02 |
| 400 | 44.6 |
| 200 | 65.78 |
| 200 | 60.59 |
| 100 | 79.24 |
| 100 | 74.05 |
| 50 | 87.8 |
| 50 | 84.85 |

| pg/mL | Average |
| --- | --- |
| 50 | 86.33 |
| 100 | 76.65 |
| 200 | 63.18 |
| 400 | 44.81 |
| 800 | 24.54 |
| 1600 | 11.92 |
| 3200 | 6.522 |

**Figure S1.2.** Example parallelism validation for assays of progesterone in faecal samples from captive, female, yearling caribou in southcentral Alaska against standards with known progesterone concentration, shown on a logarithmic scale (x axis) in a 4-parameter logistic regression model (4PL), compared with the average of the progesterone dilution in samples.

**Table S1.1.** Coefficient of variation percent (CV) results for each plate as an indicator of intra-assay variability. Plates with higher values for CV (≥10) had samples that were flagged for errors and we repeated assays on those samples. The overall mean CV for the study was 3.7%.

| **Plate Number** | **CV** |
| --- | --- |
| 1 | 2.81 |
| 2 | 5.04 |
| 3 | 4.18 |
| 4 | 7.53 |
| 5 | 2.89 |
| 6 | 5.32 |
| 7 | 4.76 |
| 8 | 6.66 |
| 9 | 3.30 |
| 10 | 4.71 |
| 11 | 7.57 |
| 12 | 4.71 |
| 13 | 5.08 |
| 14 | 6.60 |
| 15 | 7.01 |
| 16 | 5.69 |
| 17 | 6.20 |
| 18 | 6.92 |
| 19 | 6.50 |
| 20 | 8.05 |
| 21 | 4.52 |
| 22 | 3.61 |
| 23 | 4.61 |
| 24 | 6.30 |
| 25 | 3.81 |
| 26 | 6.84 |
| 27 | 6.92 |
| 28 | 7.66 |
| 29 | 10.51 |
| 30 | 10.80 |
| 31 | 7.66 |
| 32 | 5.50 |
| 33 | 7.07 |
| 34 | 6.30 |
| 35 | 5.43 |
| 36 | 9.42 |
| 37 | 5.28 |
| 38 | 8.00 |
| 39 | 4.90 |
| 40 | 7.77 |
| 41 | 9.70 |
| 42 | 5.90 |
| 43 | 7.54 |
| 44 | 4.46 |
| 45 | 7.46 |
| 46 | 10.18 |
| 47 | 31.30 |
| 48 | 39.00 |
| 49 | 4.49 |
| 50 | 5.78 |
| 51 | 9.31 |
| 52 | 5.33 |
| 53 | 9.70 |
| 54 | 9.90 |
| 55 | 12.95 |
| 56 | 7.60 |
| 57 | 10.80 |
| 58 | 8.66 |
| 59 | 7.08 |

**References**

Ajó, A. F., Hunt, K. E., Dillon, D., Uhart, M., Sironi, M., Rowntree, V., & Buck, C. L. (2022). Optimizing hormone extraction protocols for whale baleen: tackling questions of solvent:sample ratio and variation. *General and Comparative Endocrinology*, *315*, 113828.

Grotjan, H. E., & Keel, B. A. (1996). Data interpretation and quality control. In E. P. Diamandis & T. K. Christopoulos (Eds.), *Immunoassay* (pp. 51–93). Academic Press, New York, New York, USA.

Denryter, K. 2026. Influences of body fat and body protein in oestrus and reproductive potential of yearling caribou. Conservation Physiology.

**Supplementary Information 2.** Additional results.

**Table S2.1.** Results of models showing correlations between body fat, body protein, and body mass in female yearling caribou in captivity, in southcentral Alaska, in autumn 2023 and 2024.

| Model | Parameter | Coef. | SE | *t* | *P* | 95% CI |
| --- | --- | --- | --- | --- | --- | --- |
| Body fat ~ Body protein | Body protein | 3.32 | 0.55 | 6.03 | <0.001 | 2.07–4.57 |
|  | Intercept | -41.1 | 9.50 | -4.33 | 0.002 | -62.6–19.6 |
|  |  |  |  |  |  |  |
| Body fat ~ Body mass | Body mass | 0.76 | 0.10 | 7.88 | <0.001 | 0.54–0.97 |
|  | Intercept | -64.4 | 10.20 | -6.31 | <0.001 | -87.5–(-41.4) |
|  |  |  |  |  |  |  |
| Body protein ~ Body mass | Body mass | 0.22 | 0.01 | 18.55 | <0.001 | 0.19–0.24 |
|  | Intercept | -5.70 | 1.23 | -4.62 | 0.001 | -8.49–(-2.91) |

**Table S2.2.** Results of regression models for peak faecal progesterone concentration in female yearling caribou in captivity, in southcentral Alaska, in autumn 2023 and autumn–winter 2024–25, none of which were statistically significant.

| Model | Parameter | Coef. | SE | *t* | *P* | 95% CI |
| --- | --- | --- | --- | --- | --- | --- |
| Peak faecal progesterone ~ Body mass | Body mass | -20.0 | 19.5 | -1.03 | 0.330 | -64.0–24.0 |
|  | Intercept | 4468 | 2068 | 2.16 | 0.059 | -209–9146 |
|  |  |  |  |  |  |  |
| Peak faecal progesterone ~ Body fat | Body fat | -26.3 | 23.6 | -1.11 | 0.294 | -79.7–27.1 |
|  | Intercept | 2761 | 423 | 6.52 | 0.000 | 1804–3719 |
|  |  |  |  |  |  |  |
| Peak faecal progesterone ~ Body protein | Body protein | -74.9 | 90.0 | -0.83 | 0.427 | -278–129 |
|  | Intercept | 3631 | 1552 | 2.34 | 0.044 | 120–7141 |

**Table S2.3.** Results of regression models for oestrous cycle length in female yearling caribou in captivity, in southcentral Alaska, in autumn 2023 and autumn–winter 2024–25, none of which were statistically significant.

| Model | Parameter | Coef. | SE | *t* | *P* | 95% CI |
| --- | --- | --- | --- | --- | --- | --- |
| Estrous cycle length ~ Body mass | Body mass | 0.088 | 0.115 | 0.76 | 0.465 | -0.173–0.348 |
|  | Intercept | 13.3 | 12.2 | 1.09 | 0.305 | -14.4–41.0 |
|  |  |  |  |  |  |  |
| Estrous cycle length ~ Body fat | Body fat | 0.105 | 0.141 | 0.75 | 0.474 | -0.213–0.425 |
|  | Intercept | 21.0 | 2.53 | 8.28 | <0.001 | 15.2–26.7 |
|  |  |  |  |  |  |  |
| Estrous cycle length ~ Body protein | Body protein | 0.424 | .521 | 0.82 | 0.436 | -0.753–1.60 |
|  | Intercept | 15.4 | 8.98 | 1.71 | 0.121 | -4.96–35.7 |

**Figure S2.1.** Progesterone concentrations in faecal samples from *n* = 1 female yearling caribou (B) in captivity in southcentral Alaska throughout autumn–winter 2024–2025. Initiation of oestrus is exhibited by a nadir in progesterone levels and one oestrus cycle comprising the time between two consecutive nadirs. The peaks near 2,000 ng/mg progesterone in January and March are likely in error as they do not follow the typical pattern observed in confirmed oestrous cycles, and are more likely the result of sample contamination, misidentification of the animal, or mislabeling.

**Figure S2.2.** Progesterone concentrations in faecal samples from *n* = 1 female yearling caribou (C) in captivity in southcentral Alaska throughout autumn–winter 2024–2025. Initiation of oestrus is exhibited by a nadir in progesterone levels and one oestrus cycle comprising the time between two consecutive nadirs.

**Figure S2.3.** Progesterone concentrations in faecal samples from *n* = 1 female yearling caribou (D) in captivity in southcentral Alaska throughout autumn–winter 2024–2025. Initiation of oestrus is exhibited by a nadir in progesterone levels and one oestrus cycle comprising the time between two consecutive nadirs.

**Figure S2.4.** Progesterone concentrations in faecal samples from *n* = 1 female yearling caribou (F) in captivity in southcentral Alaska in autumn 2023. Initiation of oestrus is exhibited by a nadir in progesterone levels and one oestrus cycle comprising the time between two consecutive nadirs.

**Figure S2.5.** Progesterone concentrations in faecal samples from *n* = 1 female yearling caribou (H) in captivity in southcentral Alaska throughout autumn–winter 2024–2025. Initiation of oestrus is exhibited by a nadir in progesterone levels and one oestrus cycle comprising the time between two consecutive nadirs. The peak near 3,000 ng/mg progesterone in early March is likely an error as it does not follow the typical pattern observed in confirmed oestrous cycles, and is more likely the result of sample contamination, misidentification of the animal, or mislabeling.

**Figure S2.6.** Progesterone concentrations in faecal samples from *n* = 1 female yearling caribou (LE) in captivity in southcentral Alaska throughout autumn–winter 2024–2025. Initiation of oestrus is exhibited by a nadir in progesterone levels and one oestrus cycle comprising the time between two consecutive nadirs.

**Figure S2.7.** Progesterone concentrations in faecal samples from *n* = 1 female yearling caribou (LU) in captivity in southcentral Alaska throughout autumn–winter 2024–2025. Initiation of oestrus is exhibited by a nadir in progesterone levels and one oestrus cycle comprising the time between two consecutive nadirs.

**Figure S2.8.** Progesterone concentrations in faecal samples from *n* = 1 female yearling caribou (O) in captivity in southcentral Alaska in autumn 2023. Initiation of oestrus is exhibited by a nadir in progesterone levels and one oestrus cycle comprising the time between two consecutive nadirs.

 **Figure S2.9.** Progesterone concentrations in faecal samples from *n* = 1 female yearling caribou (S) in captivity in southcentral Alaska in autumn 2023. Initiation of oestrus is exhibited by a nadir in progesterone levels and one oestrus cycle comprising the time between two consecutive nadirs.

**Figure S2.10.** Progesterone concentrations in faecal samples from *n* = 1 female yearling caribou (Z) in captivity in southcentral Alaska throughout autumn–winter 2024–2025. Initiation of oestrus is exhibited by a nadir in progesterone levels and one oestrus cycle comprising the time between two consecutive nadirs.

**Table S2.4.** Overwinter change in ingesta-free body fat (IFBF; %) and body protein (kg) of female yearling caribou in captivity, in southcentral Alaska, in autumn 2023 and 2024.

| Animal ID | Overwinter IFBF change | Overwinter protein change |
| --- | --- | --- |
| B | 3.8 | -0.8 |
| C | -0.5 | -2.5 |
| D | -7.1 | -1.3 |
| F | -5.9 | 0.3 |
| H | -2.3 | -0.2 |
| LE | -1.3 | -0.6 |
| LU | -1.3 | -3.1 |
| O | -24.5 | -3.9 |
| S | -14.8 | -2.3 |
| X | -7.3 | -1.6 |
| Z | 1.9 | -3.3 |

Denryter, K. 2026. Influences of body fat and body protein in oestrus and reproductive potential of yearling caribou. Conservation Physiology.

**Supplemental Information 3.** Additional results.

When I removed the two largest females from analyses of number of oestrous cycles relative to body fat, to evaluate whether these points were overly influential, the regression was no longer significant (F(1, 4) = 0.06, P = 0.819, r^2^ = 0.015, adj. r^2^ = -0.232) and the increase in the number of oestrous cycles per 1 percentage point increase in body fat was smaller (0.1 versus 0.3). However, it also is worth recognizing that there was much more variation among the thinner animals, and there was a strong relationship for 4 individuals on the regression line. When I ran the regression without the individuals falling outside the 95% CIs for all data, the result was a highly significant regression equation (F(1, 2) = 586.65, P = 0.002, r^2^ = 0.997, adj. r^2^ = 0.995) and an increase of 0.2 oestrous cycles per 1 percentage point increase in body fat in autumn.
